# Therapeutic potential of cannabinoids in neurological conditions: a systematic review of clinical trials

**DOI:** 10.3389/fphar.2025.1521792

**Published:** 2025-02-06

**Authors:** Alqassem Y. Hakami, Fahad S. Alshehri

**Affiliations:** ^1^ College of Medicine, King Saud Bin Abdulaziz University for Health Sciences, Jeddah, Saudi Arabia; ^2^ King Abdullah International Medical Research Center, Jeddah, Saudi Arabia; ^3^ Department of Pharmacology and Toxicology, College of Pharmacy, Umm Al-Qura University, Makkah, Saudi Arabia

**Keywords:** neurodegenerative, neurological, tetrahydrocannabinol, cannabidiol, clinical trials

## Abstract

**Overview:**

Cannabinoids have gained increasing attention for their therapeutic potential in treating several neurological conditions, including neurodegenerative diseases, chronic pain, and epilepsy. This review aims to assess the current clinical trials investigating cannabinoids, primarily Tetrahydrocannabinol and Cannabidiol, for neurological disorders. This review will aim to highlight the efficacy, safety, and outcome measures used in these trials.

**Methods:**

Clinical trials were identified using ClinicalTrials.gov, focusing on studies that examined the effects of cannabinoids in treating neurological conditions. All trials that fulfilled the following criteria were included: Phase 1–4, focused on cannabinoids as primary intervention, and measured relevant outcomes such as pain relief, cognitive function, or spasticity reduction. Data on conditions, interventions, primary and secondary outcomes, and trial phases were extracted and analysed.

**Results:**

A total of 47 clinical trials were identified, including different neurological conditions. The most frequently studied conditions were Multiple Sclerosis, Fibromyalgia, and Parkinson’s Disease. Most trials were in Phase 2, with the primary outcome measures focused on pain management, spasticity, and cognitive function. Secondary outcomes included safety and tolerability measures.

**Conclusion:**

The review highlights the broad therapeutic potential of cannabinoids in neurology, with promising results in symptom management for conditions like Multiple Sclerosis and Fibromyalgia. However, the lack of standardized study protocols, dosing, and outcome measures presents challenges for broader clinical implementation.

**Systematic Review Registration:**

clinicatrials.gov.

## Introduction

In recent years, the therapeutic applications of cannabinoids from Cannabis sativa, a plant belonging to the Cannabaceae family, have significant interest. Cannabinoids, the principal compounds of this species, are predominantly classified into psychoactive and non-psychoactive types (Cristino et al., 2020; Mechoulam, 2019). The most studied cannabinoids, Δ9-tetrahydrocannabinol (THC) and cannabidiol (CBD), are phytocannabinoids derived from the cannabis plant (Andre et al., 2016; Elmes et al., 2015). While THC is the psychoactive component of cannabis, CBD is non-psychoactive and has been widely studied for its potential therapeutic benefits (Scuderi et al., 2009). These compounds interact with the endocannabinoid system in humans, which plays a crucial role in regulating various physiological processes including pain sensation, immune response, and neuroprotection (Lowe et al., 2021). This system the commonly known G-protein-coupled receptor. Cannabinoid receptor (CBR1 and CBR2); and range of endogenous ligands and enzymes responsible for the synthesis and degradation of cannabinoids, emphasizing its complexity and significance in neuropharmacology (Keimpema et al., 2014; Lu and Mackie, 2021).

The endocannabinoid system is not limited to its two primary G-protein-coupled receptors, CBR1 and CBR2. It also includes a network of endogenous cannabinoids, such as anandamide and 2-arachidonoylglycerol, and enzymes like fatty acid amide hydrolase (FAAH) and monoacylglycerol lipase (MAGL), which synthesize and degrade these endocannabinoids. These components are crucial for the modulation of various physiological processes (Kilaru and Chapman, 2020). Importantly, cannabinoids interact with the endocannabinoid system to modulate neurotransmission and neuroinflammation, central mechanisms in the development and persistence of neuropathic pain (Guindon and Hohmann, 2009a; Woodhams et al., 2015). By binding to CBRs in the nervous system, these compounds can inhibit the release of neurotransmitters and pain signaling pathways, offering potential relief in conditions characterized by chronic pain and hyperalgesia (Finn et al., 2021; Mlost et al., 2019a). This interaction also suggests a broader role in neuroprotection and neuroplasticity, which could underlie their therapeutic benefits across a neuropathic disorders (Xu and Chen, 2015).

CBR1 is predominantly found in the brain and are involved in regulating neurotransmitter release (Busquets-Garcia et al., 2018), while CBR2 are mainly expressed in immune cells and peripheral tissues, where they modulate inflammatory processes (Turcotte et al., 2016). The endocannabinoid system presents a potential for therapeutic interventions targeting neurological disorders, where dysregulation of the endocannabinoid system has been implicated. The potential therapeutic applications of cannabinoids extend across a range of neurological conditions, including neurodegenerative diseases such as Alzheimer’s disease (Benito et al., 2007), Parkinson’s disease (Di Filippo et al., 2008), and Huntington’s disease (Pazos et al., 2008), and multiple sclerosis (MS) (Chiurchiu et al., 2018), epilepsy (Kwan Cheung et al., 2019), and chronic pain conditions like neuropathy (Maldonado et al., 2016). With the increasing prevalence of these conditions and the limited efficacy of existing treatments (Feigin et al., 2020), the exploration of cannabinoids as novel therapeutic agents has accelerated. Clinical trials have played a crucial role in evaluating the safety, efficacy, and mechanisms of action of cannabinoids in treating these neurological conditions.

Cannabinoids, particularly THC and CBD, have been explored for their ability to manage spasticity, neuropathic pain, and bladder dysfunction in MS patients (Baker et al., 2000; Fontelles and García, 2008; Zajicek and Apostu, 2011). Sativex, an oromucosal spray containing both THC and CBD, has been approved in several countries for the treatment of spasticity in MS (Giacoppo et al., 2017). Clinical trials have shown Sativex efficacy in reducing spasticity and improving quality of life in patients with refractory symptoms (Nurmikko et al., 2007; Vermersch, 2011). The underlying mechanisms of cannabinoids in MS appear to involve the modulation of immune cell activity, reduction of pro-inflammatory cytokine release, and preservation of neuronal integrity (Chiurchiu et al., 2018; Khan et al., 2022). In addition, another CBD-based oral product was approved by the United States Food and Drug Administration (USFDA) for the treatment of Dravet syndrome and Lennox-Gastaut syndrome (Wechsler et al., 2024; Laux et al., 2019), two rare and severe forms of childhood epilepsy (Sullivan et al., 2024). Cannabidiol success was supported by multiple randomized, placebo-controlled clinical trials that confirmed a significant reduction in seizure frequency in patients receiving CBD compared to those receiving placebo (Lattanzi et al., 2021; Miller et al., 2020; Patel et al., 2021). The exact mechanism by which cannabinoids reduce seizures is not fully understood, but it is believed to involve the modulation of voltage-gated ion channels, inhibition of glutamate release (Rosenberg et al., 2017), and increase the inhibitory GABAergic neurotransmission (Ruffolo et al., 2022). Therefore, restore the balance between excitatory and inhibitory signalling in the brain, which is often disrupted in epilepsy.

Chronic pain, particularly neuropathic pain, is one of the most challenging conditions to manage in the neurology field (Finnerup et al., 2021; Bernetti et al., 2021). Neuropathic pain is caused by damage to the nervous system and is often resistant to conventional analgesics, including opioids (Hange et al., 2022). The endocannabinoid system is thought to play a central role in modulating pain pathways (Greco et al., 2022), making cannabinoids a potential therapeutic option for patients with neuropathic pain. Clinical trials investigating cannabinoids in neuropathic pain have revealed contrary outcomes, with some studies reporting significant pain relief (Campos et al., 2021; Cumenal et al., 2021), while others showing less significant outcomes in regards to pain management (Selvarajah et al., 2010). However, cannabinoids are generally well-tolerated and offer a favourable safety profile compared to opioids (Pisani et al., 2021), making them an important alternative for patients seeking non-opioid pain management options.

Regardless of advancements in neurological therapeutics, significant limitations in the current treatment, particularly regarding efficacy and safety profiles. Many conventional therapies provide insufficient relief or pose substantial side effects, such as cognitive dulling, dependency, or even exacerbation of symptoms in long-term use. This highlights a clinical need for alternative therapeutic strategies, such as cannabinoids, which have shown in early trials to address these gaps effectively. This review examines the therapeutic potential of cannabinoids in managing cognition, pain, and spasticity in neurological conditions, with a strong focus on safety and tolerability. It aims to guide future research and clinical practice on how these compounds address specific symptoms and underpin safe usage.

## Methodology

### Data source

This study used a secondary data analysis approach to evaluate the therapeutic potential of cannabinoids, specifically focusing on clinical trials involving CBD and THC for neurological conditions. The data were obtained from publicly available records on ClinicalTrials.gov, with trials being filtered based on their completion status and relevance to neurological disorders.

### Data collection

**FIGURE 1:**
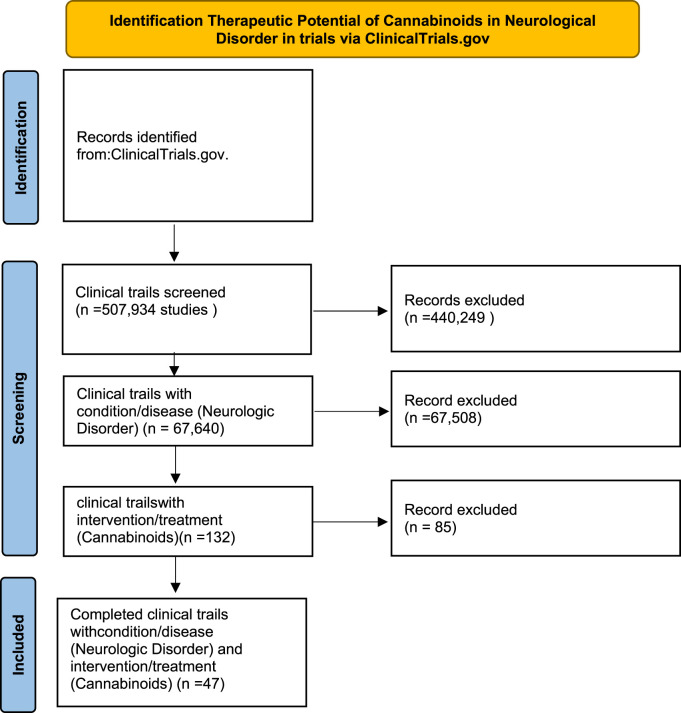
Methods used to identify therapeutic potential of cannabinoids in neurological disorder.

The methodology for identifying and selecting clinical trials involved a multi-step process, as illustrated in (Figure 1). Initial Screening: A total of 507,934 clinical trials were initially identified from ClinicalTrials.gov. After excluding irrelevant records, 67,640 trials focusing on neurological disorders were retained for further analysis. Cannabinoid-Specific Trials: From this, trials involving cannabinoid interventions were identified, leading to 132 trials. Further exclusions based on incomplete or irrelevant data, total of 47 completed clinical trials were included in the final analysis. The search on ClinicalTrials.gov initially identified 507,934 clinical trials. We applied specific search terms including “cannabinoids”, “neurological disorders”, and names of specific conditions like “Multiple Sclerosis” and “Parkinson’s Disease”. We further refined the search by setting filters for trial status to “completed”, phases to include “Phase 1-4”, and interventions specifically focusing on cannabinoids. We excluded trials that did not meet our inclusion criteria: studies that were not completed as of the data extraction date, those not focusing on cannabinoids as the primary intervention or lacking clear primary outcome measures related to the efficacy and safety in neurological conditions.

### Comparative analysis

In our systematic review, we analysed 47 clinical trials on cannabinoids for neurological conditions, focusing particularly on 13 trials involving CBD and THC. These compounds, making up about 27% of our study sample, were chosen for their significant therapeutic potential and regulatory relevance. This focus allowed us to deeply explore their efficacy, safety, and mechanisms of action, while comparisons with other cannabinoid studies enhanced our understanding of CBD and THC’s specific effects. Following the identification of relevant trials, this review concentrated on comparing CBD and THC outcomes. The comparison includes the following criteria: the total number of clinical trials conducted for each cannabinoid, the specific neurological conditions they targeted, the distribution of trials across different clinical research phases (Phase 1, Phase 2, and Phase 3), and the primary outcome measures, such as pain relief, tremor reduction, and safety evaluations.

### Data analysis

Descriptive statistics were used to summarize the key characteristics of the trials, and a table was generated to highlight the differences between CBD and THC trials. This comparison allowed for a clear understanding of the therapeutic focus and outcomes of each cannabinoid in neurological disorders. The results of the analysis were presented in a table format to facilitate the easy comparison. We assessed the risk of bias for each included study using the Cochrane Collaboration’s tool, focusing on randomization, blinding, completeness of outcome data, and selective reporting. Each domain was rated as “Low Risk”, “High Risk”, or “Unclear Risk” based on the criteria established by the Cochrane Handbook.

## Results

This study analysed total of 47 completed clinical trials investigating the therapeutic potential of cannabinoids. While our study reviewed a broad range of clinical trials on cannabinoids, we specifically focused on those trials that primarily investigated CBD and THC. Although other cannabinoids such as Nabilone and Dronabinol were present in the dataset, our analysis cantered on understanding the effects and outcomes related to CBD and THC in treating neurological conditions. The 47 clinical trials revealed that the majority were in Phase 2, constituting a significant proportion of the dataset. The importance of Phase 2 trials is particularly relevant as these studies are designed to provide preliminary evidence on efficacy and a more precise assessment of safety, which are critical for subsequent larger-scale Phase 3 trials. This phase distribution underscores the developmental stage of cannabinoid use in neurological conditions and its potential readiness for more advanced clinical testing. Furthermore, the outcome measures across these trials primarily focused on efficacy in symptom management and safety profiles. For instance, pain management, spasticity reduction, and cognitive function improvements were commonly reported. The trials were selected based on their focus on cannabinoids and neurological disorders, and their characteristics are summarized in Table 1.

**TABLE 1:** Summary of clinical trials. This table presents the key characteristics of 47 clinical trials that focused on the use of cannabinoids for treating various neurological conditions.

Characteristic	Details	Percentage (%)
Total Studies	47	100%
Most Common Conditions	Psychomotor Impairment	-
	Parkinson’s Disease	-
	Fibromyalgia	-
Most Common Interventions	Nabilone	-
	Dronabinol	-
	CBD Oil	-
	THC	-
Study Phases	Phase not available (13 studies)	27.6%
	Phase 1 (3 studies)	6.3%
	Phase 1 & 2 (4 studies)	8.5%
	Phase 2 (18 studies)	38.3%
	Phase 2 & 3 (1 study)	2.1%
	Phase 3 (7 studies),	14.9%
	Phase 4 (1 study)	2.1%
Study Types	Interventional (45 studies)	95.7%
	Observational (2 studies)	4.3%
Age Categories	Adults and Older Adults (38 studies)	80.9%
	Adults only (6 studies)	12.8%
	Children & Adults (2 studies)	4.3%
Dosage	Varies by study (e.g., 10 mg to 600 mg per day for CBD)	-
Duration of Treatment	Ranges from 4 weeks to 2 years, depending on the study	-
Route of Administration	Oral, Sublingual, Topical, Inhalation	-

### Total number of trials

The analysis included 47 clinical trials, only six trials involving CBD and seven trials involving THC. These trials varied in their conditions of focus and study design, with several trials investigating combinations of both cannabinoids to evaluate potential synergistic effects. The remaining studies involved other cannabinoid compounds like Nabilone and Dronabinol. Despite the variation in trials, both CBD and THC emerged as the key cannabinoids explored for their therapeutic benefits in neurological disorders.

### Conditions studied

The conditions studied in CBD and THC trials largely overlapped, focusing on neurological and pain-related conditions, though the scope of THC trials was broader. CBD trials predominantly targeted conditions such as temporomandibular joint (TMJ) disorder, Huntington’s disease, migraine, essential tremor, and neuropathic pain. In contrast, THC trials covered a wider spectrum of conditions, including behavioral disturbances and neurodegenerative diseases like dementia (Alzheimer’s and vascular types), alongside more typical conditions like essential tremor and neuropathic pain. This broader focus suggests that THC may offer therapeutic benefits beyond pain relief, extending into cognitive and behavioral symptoms. A detailed comparison is provided in Table 2.

**TABLE 2:** CBD vs. THC trials comparison. This table compares the clinical trials that investigated CBD (Cannabidiol) versus THC (Tetrahydrocannabinol).

Aspect	CBD	THC	Details
Total Trials	6	7	Total number of trials involving each compound
Percentage of Total	(12.7%)	(14.9%)	Proportion of trials out of total studied
Conditions Studied	TMJ Disorder	Huntington’s Disease	Specific conditions targeted by each cannabinoid
	Huntington’s Disease	Dementia	
	Migraine	Migraine	
	Essential Tremor	Essential Tremor	
	Neuropathic Pain	Neuropathic Pain	
Study Phases	Phase 2: 3 studies	Phase 2: 5 studies	Phases of studies involving each compound
	Phase 1/2: 1 study	Phase 1/2: 1 study	
Primary Outcome Measures	Pain severity	Pain intensity	Key outcomes measured in studies involving each compound
	Headache pain relief	Headache pain relief	
	Change in baseline pain (NRS)	Tremors mean amplitude (digital spirography)	

### Study phases

Both CBD and THC trials were largely concentrated in Phase 2, a critical stage where the efficacy and side effects of the treatment are tested in larger groups. Among the CBD trials, three were in Phase 2 and 1 was a combined Phase 1/2 trial. Similarly, THC trials had five studies in Phase 2, with an additional Phase 1/2 trial. This reflects an emphasis on mid-stage clinical testing for both cannabinoids, as researchers aim to assess the therapeutic potential and safety of cannabinoids for neurological disorders before moving to larger, late-stage trials.

### Primary outcome measures

**FIGURE 2:**
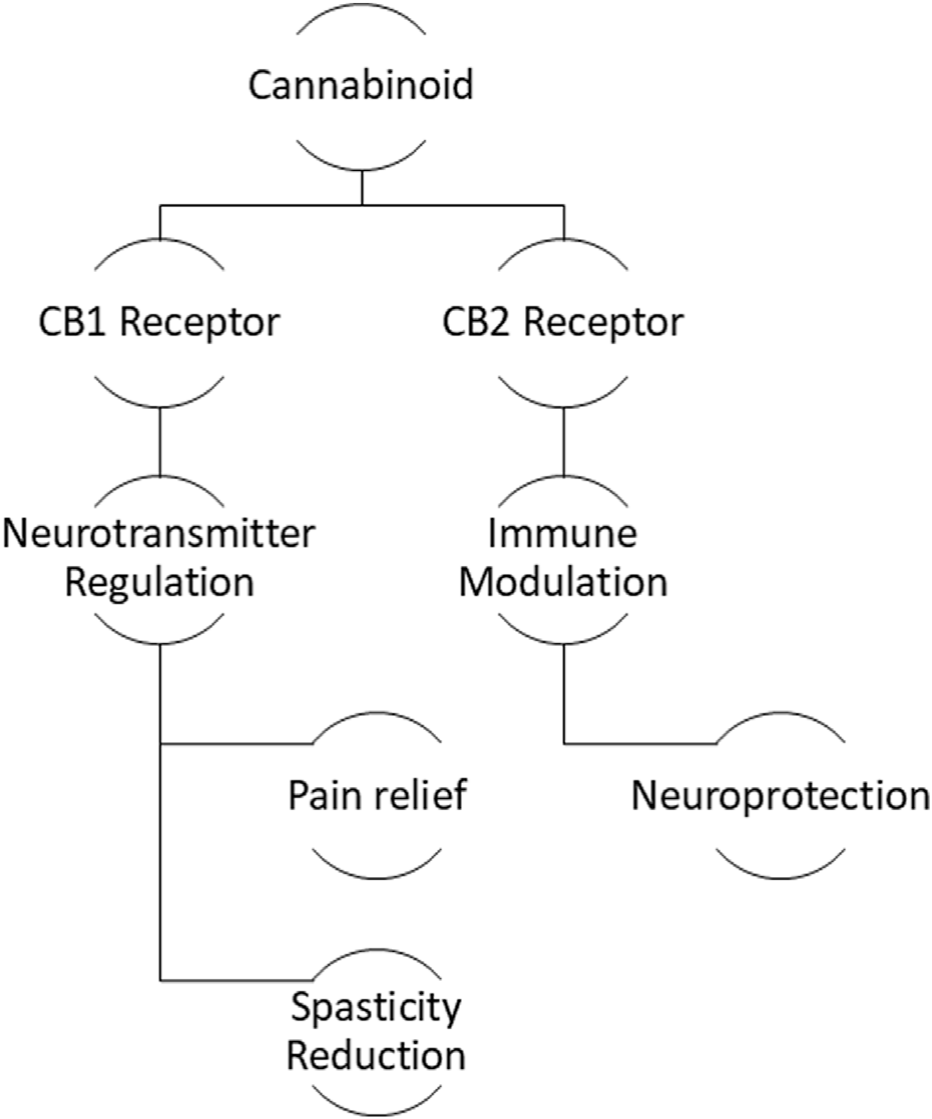
Mechanism of cannabinoid therapeutics: Interaction with CB1 and CB2 receptors leading to neuroprotection and symptom management.

The primary outcomes measured in these trials revolved primarily around pain management, but there were also differences in focus between CBD and THC. CBD trials predominantly focused on pain relief, particularly in conditions such as migraine and TMJ disorder, where pain severity and functional limitations were key outcomes. Outcome measures such as changes in baseline pain (using numeric rating scales) and self-reported pain severity were common across these trials. On the other hand, THC trials also focused on pain management but extended into other areas such as motor symptoms and cognitive/behavioural assessments. For example, tremor amplitude was measured in patients with essential tremor, while behavioural disturbances were evaluated in dementia patients. These trials aimed to assess not only symptom relief but also improvements in motor and cognitive functioning, illustrating THC’s broader therapeutic potential in managing neurological symptoms beyond pain, Figure 2.

### Safety and adverse effects

Both CBD and THC trials monitored for adverse effects, with the majority reporting mild to moderate side effects. Common side effects across both types of trials included dizziness, dry mouth, and fatigue. These side effects were generally well-tolerated, and no severe adverse events were reported in most trials. This suggests that both CBD and THC can be considered relatively safe when administered under controlled clinical conditions, though more extensive post-market trials (Phase 4) are necessary to assess long-term safety.

Our risk-of-bias assessment revealed that while randomization procedures were generally well-handled across the studies (Low Risk), the majority lacked sufficient blinding (High Risk), raising concerns about potential performance and detection biases. Additionally, the high risk of outcome reporting bias suggests a need for more transparent and complete reporting of trial results as shown in supplements Table 3.

**TABLE 3:** Cochrane collaboration risk-of-bias assessment for included studies.

Randomization	Blinding	Outcome reporting	Selective reporting	Study title
Low Risk	High Risk	High Risk	Low Risk	Comparison of Cannabinoids to Placebo in Management of TMJ Pain and Myofascial Pain in the TMJ Region
Low Risk	High Risk	High Risk	Low Risk	Experimental Medicine in ADHD - Cannabinoids
Low Risk	High Risk	High Risk	Low Risk	Neuroprotection by Cannabinoids in Huntington’s Disease
Low Risk	High Risk	High Risk	Low Risk	CANNAbinoids in the Treatment of TICS (CANNA-TICS)
Low Risk	High Risk	High Risk	Low Risk	Radicle Rest: A Study of Cannabinoids on Sleep and Health Outcomes
Unclear Risk	High Risk	Low Risk	Low Risk	Nabilone for Non-motor Symptoms in Parkinson’s Disease
Low Risk	High Risk	Low Risk	Low Risk	Nabilone for Non-motor Symptoms in Parkinson’s Disease
Low Risk	High Risk	High Risk	Low Risk	Trial of Dronabinol Adjunctive Treatment of Agitation in Alzheimer’s Disease
Low Risk	High Risk	High Risk	Low Risk	Cannabinol Use in Patients With Insomnia Disorder
Low Risk	High Risk	High Risk	Low Risk	Full-spectrum Medical Cannabis for Treatment of Spasticity in Patients With Severe Forms of Cerebral Palsy
Low Risk	High Risk	High Risk	Low Risk	Orexigenic Therapy With Delta-9-tetrahydrocannabinol in Advanced Cancer Patients With Chemosensory Abnormalities - a Pilot Study
Unclear Risk	High Risk	High Risk	Low Risk	Cannabinoid Therapy for Pediatric Epilepsy
Unclear Risk	High Risk	High Risk	Low Risk	Pain Research: Innovative Strategies With Marijuana
Low Risk	High Risk	High Risk	Low Risk	BX-1 in Spasticity Due to Multiple Sclerosis
Low Risk	High Risk	High Risk	Low Risk	Study to Assess the Safety, Tolerability, and Effects of CHI-202 to Support Recovery From Physical Activity
Low Risk	High Risk	High Risk	Low Risk	Safety and Efficacy on Spasticity Symptoms of a Cannabis Sativa Extract in Motor Neuron Disease
Low Risk	High Risk	High Risk	Low Risk	Effect of Cannabinoids on Spasticity and Neuropathic Pain in Spinal Cord Injured Persons
Low Risk	High Risk	High Risk	Low Risk	Nabilone Versus Amitriptyline in Improving Quality of Sleep in Patients With Fibromyalgia
Low Risk	High Risk	High Risk	Low Risk	A Trial Assessing the Effect of Nabilone on Pain and Quality of Life in Patients With Fibromyalgia
Low Risk	High Risk	High Risk	Low Risk	Diagnosis and Therapy of Vulnerable Atherosclerotic Plaque
Unclear Risk	High Risk	High Risk	Low Risk	An Observational Post-Marketing Safety Registry of Sativex آ^®^
Unclear Risk	High Risk	Low Risk	Low Risk	Efficacy of a Therapeutic Combination of Dronabinol and PEA for Tourette Syndrome
Unclear Risk	High Risk	High Risk	Low Risk	Efficacy of Palmitoylethanolamide-polydatin Combination on Chronic Pelvic Pain in Patients With Endometriosis
Low Risk	High Risk	High Risk	Low Risk	Trial to Evaluate Efficacy and Safety of Lenabasum in Dermatomyositis
Low Risk	High Risk	Low Risk	Low Risk	Trial to Evaluate the Effect of Nabiximols Oromucosal Spray on Clinical Measures of Spasticity in Participants With Multiple Sclerosis
Low Risk	High Risk	High Risk	Low Risk	Delta-THC in Dementia
Low Risk	High Risk	High Risk	Low Risk	Combined Alcohol and Cannabis Effects on Skills of Young Drivers
Low Risk	High Risk	High Risk	Low Risk	Nabilone for the Treatment of Phantom Limb Pain
Low Risk	High Risk	High Risk	Low Risk	A Study Evaluating the Effectiveness of PEA Compared to Placebo for Reducing Pain Severity and Duration of Migraines
Low Risk	High Risk	Low Risk	Low Risk	Cannabis Effects on Driving-related Skills of Young Drivers
Low Risk	High Risk	High Risk	Low Risk	Efficacy of Inhaled Cannabis for Acute Migraine Treatment
Low Risk	High Risk	High Risk	Low Risk	Efficacy and Safety of the Pain Relieving Effect of Dronabinol in Central Neuropathic Pain Related to Multiple Sclerosis
Low Risk	High Risk	High Risk	Low Risk	9”δ- THC (Namisol آ^®^ ) in Chronic Pancreatitis Patients Suffering From Persistent Abdominal Pain
Low Risk	High Risk	High Risk	High Risk	Study to Evaluate the Efficacy and Safety of Dronabinol Metered Dose Inhaler (MDI) in Acute Treatment of Migraine Headache
Low Risk	High Risk	High Risk	Low Risk	Effects of Rimonabant on Walking Abilities in Incomplete Spinal Cord Injury
Low Risk	High Risk	Low Risk	Low Risk	Trial of Cannabis for Essential Tremor
Low Risk	High Risk	High Risk	Low Risk	Pain Inflammation and Cannabis in HIV
Low Risk	High Risk	High Risk	Low Risk	Acetylcholine Receptors From Human Muscles as Pharmacological Target for ALS
Low Risk	High Risk	High Risk	Low Risk	Randomized Placebo-Controlled Crossover Trial With THC (Delta 9-Tetrahydrocannabinol) for the Treatment of Cramps in Amyotrophic Lateral Sclerosis (ALS)
Low Risk	High Risk	Low Risk	Low Risk	Trial of Dronabinol and Vaporized Cannabis in Chronic Low Back Pain
Low Risk	High Risk	High Risk	Low Risk	Delta-THC in Behavioral Disturbances in Dementia
Low Risk	High Risk	High Risk	Low Risk	Efficacy Study of 9”δ- THC to Treat Chronic Abdominal Pain
Low Risk	High Risk	Low Risk	Low Risk	Safety and Efficacy Study of Dronabinol to Treat Obstructive Sleep Apnea
Low Risk	High Risk	Low Risk	Low Risk	Study for Efficacy and Dose Escalation of AD313 + Atomoxetine (SEED)
Low Risk	High Risk	High Risk	Low Risk	Micronized and Ultramicronized Palmitoylethanolamide in Fibromyalgia Patients
Unclear Risk	High Risk	Low Risk	Low Risk	A Pilot Study of Dronabinol for Adult Patients With Primary Gliomas
Unclear Risk	High Risk	High Risk	Low Risk	Randomized Double Blind Cross Over Study for Nabilone in Spasticity in Spinal Cord Injury Persons

## Discussion

Our systematic review examined 47 clinical trials on cannabinoids in neurological conditions, with a focused analysis on the 13 trials involving CBD and THC, which represent about 27% of our sample. These trials are highlighted due to their significant insights into the therapeutic potential and regulatory considerations of CBD and THC. Including other cannabinoids provides a comparative context, enhancing our understanding of the unique effects of CBD and THC in neurological therapies. The results of this study highlight the growing body of evidence surrounding the therapeutic potential of cannabinoids, particularly CBD and THC, in treating a range of neurological disorders. The clinical trials reviewed in this analysis provide insights into how cannabinoids can be used to manage symptoms such as chronic pain, motor dysfunction, cognitive impairment, and behavioural disturbances. The diversity of conditions studied, from neurodegenerative diseases like Huntington’s and Alzheimer’s to more common disorders like migraine and neuropathic pain, underscores the wide-ranging potential of cannabinoids in neurology. However, while the therapeutic potential of these compounds is evident, the variability in trial design, outcome measures, and focus between CBD and THC requires careful consideration to understand the specific benefits and limitations of each cannabinoid.

One of the primary areas where cannabinoids have shown potential outcomes is in the management of chronic pain, which is a crucial mark of many neurological disorders. Both CBD and THC have been investigated for their analgesic properties, with most trials measuring pain severity and symptom relief as key outcomes. CBD trials, in particular, focused heavily on pain management in conditions such as TMJ disorder, migraine, and neuropathic pain, with outcomes often measured through patient-reported pain scores and functional limitations. The analgesic effects of CBD are presumed to be a result of its ability to modulate the endocannabinoid system and influence neurotransmitter release, particularly through the regulation of serotonin and dopamine pathways, which play critical roles in pain perception (Mlost et al., 2019b; Guindon and Hohmann, 2009b; Salaga et al., 2019). However, the exact mechanisms by which CBD produce its effects remain an active area of research, and the current trials provide only preliminary evidence for its use in chronic pain management. Additional studies with larger sample sizes and more rigorous designs are necessary to confirm these findings and to explore optimal dosing strategies for different types of pain.

In contrast, THC trials not only explored pain management but also extended into the treatment of motor and cognitive dysfunction, particularly in neurodegenerative diseases like Huntington’s and Alzheimer’s disease. THC’s effects on the endocannabinoid system involve its action as a partial agonist at CBR1 and CBR2 in the brain and peripheral nervous system. By activating these receptors, THC can influence motor control and reduce tremor severity, making it a potential candidate for treating conditions such as essential tremor and Parkinson’s disease (Buhmann et al., 2019). THC also has psychoactive effects, which can be both beneficial and limiting. In diseases like Alzheimer’s, THC has been shown to reduce agitation and behavioural disturbances, potentially providing relief for patients suffering from cognitive decline (Outen et al., 2021). However, its psychoactive properties raise concerns about side effects such as cognitive impairment and dizziness (Solimini et al., 2017), particularly in older adults who may already be vulnerable to cognitive issues (Beedham et al., 2020). This dual nature of THC providing both therapeutic benefits and psychoactive risks requires careful patient selection and close monitoring in clinical practice.

Another important aspect of this analysis is the difference in study phases between CBD and THC trials. The majority of the trials for both cannabinoids were concentrated in Phase 2, which is typically designed to assess efficacy and side effects in a larger cohort than Phase 1 trials. While Phase 2 studies provide valuable information on the potential benefits of cannabinoids, the lack of Phase 4 studies in the dataset is a significant limitation. Phase 4, or post-marketing surveillance, trials are essential for understanding the long-term effects and safety of cannabinoids when used in larger, more diverse populations outside the controlled environment of clinical trials (Resnik, 2007; Gough, 2005; Lunghi et al., 2022). The absence of Phase 4 data limits our ability to make definitive conclusions about the widespread use of CBD and THC in routine clinical practice. One of the key findings of this analysis is the comprehensive therapeutic scope of THC compared to CBD. While both cannabinoids are effective in managing pain, THC trials explored additional conditions such as dementia, behavioural disturbances, and motor disorders. The inclusion of conditions like Alzheimer’s disease in THC trials highlights its potential beyond pain relief, particularly in addressing the neuropsychiatric symptoms of neurodegenerative diseases (Cummings, 2021). This comprehensive range of applications for THC suggests that it may have a more useful role in neurology than CBD, although the psychoactive effects of THC remain a significant concern. However, CBD is often seen as a safer alternative due to its non-psychoactive nature, making it more suitable for patients who are sensitive to the mind-altering effects of THC (Benson, 2019; Stella, 2023). However, the narrow focus of CBD trials on pain and functional limitations may indicate that its therapeutic benefits are more restricted compared to THC, at least in the current body of evidence.

While THC’s psychoactive properties can limit its usability, strategies can be done to reduce these effects in clinical practice. Dose adjustments, based on patient response and tolerance, and the co-administration of CBD, which may counteract some of THC’s psychoactive effects, are viable approaches (Schecter and Cyr, 2022a; Kitdumrongthum and Trachootham, 2023). Furthermore, developing formulations that balance THC and CBD concentrations could influence the associated effect, potentially enhancing therapeutic outcomes while minimizing adverse effects (Schecter and Cyr, 2022b). Detailed guidelines for these strategies should be explored and defined through clinical research to ensure safe and effective use of cannabinoids in treating neurological conditions.

The safety profiles of both CBD and THC, as reported in these trials, suggest that both compounds are generally well-tolerated, with mild to moderate side effects being the most commonly reported issues. Dizziness, fatigue, and dry mouth were frequently observed, but these side effects did not appear to result in significant discontinuation rates or severe adverse events. This indicates that cannabinoids can be safely administered to patients with neurological disorders, provided that appropriate dosing and monitoring are in place. However, the limited number of trials and the absence of long-term safety data remain barriers to widespread clinical implementation. As the use of cannabinoids continues to grow, it will be critical to establish comprehensive safety guidelines and dosing protocols to ensure that the benefits of these therapies outweigh the risks for all patient populations.

As a limitation of our findings, future research should include multiple trial registries, such as the WHO’s International Clinical Trials Registry Platform (WHO ICTRP). Additionally, the variability in cannabinoid formulations and dosages across studies may affect the consistency and generalizability of results. Furthermore, many cannabinoid trials are short-term; long-term studies are needed to fully understand the safety and efficacy of cannabinoids for chronic neurological conditions. The potential biases identified through our risk-of-bias assessment may limit the reliability of our findings. Particularly, the high risk associated with unblinded studies and incomplete outcome reporting could have skewed the efficacy and safety profiles of cannabinoids reported in this review. The inclusion of studies with accurate methodological designs, including proper blinding and randomization, is also crucial to minimize bias. Lastly, potential publication biases and the varying legal and ethical landscapes surrounding cannabinoid use across countries must be considered, as these factors could significantly influence research outcomes and their interpretation. While our review confirms the therapeutic potential of cannabinoids for neurological conditions, the lack of standardized protocols and dosing regimens across the studies is a significant challenge. This variability can lead to inconsistent outcomes and complicates the determination of optimal therapeutic doses, potentially skewing the true effects of cannabinoids. These issues highlight the urgent need for future research to develop clear, evidence-based guidelines for cannabinoid treatment. Establishing standardized dosing protocols would not only improve the reliability of research findings but also facilitate their clinical application, enhancing patient safety and therapeutic outcomes.

## Conclusion

The results of this analysis showed that both CBD and THC have significant potential as therapeutic agents for neurological disorders, particularly in managing pain, motor dysfunction, and behavioural disturbances. However, their different pharmacological profiles and side effect risks mean that each cannabinoid may be better suited to different patient populations and conditions. While THC’s broader range of applications in cognitive and motor symptoms positions it as a more multipurpose treatment option, the psychoactive risks associated with its use should not be ignored. On the other hand, CBD’s safety and non-psychoactive nature make it more preferred option for managing chronic pain, but its therapeutic benefits may be more limited. Future research should focus on addressing the gaps in long-term safety and efficacy data, as well as exploring the full potential of lesser-known cannabinoids and combination therapies to further enhance the treatment of neurological disorders.

## Data Availability

The original contributions presented in the study are included in the article/supplementary material, further inquiries can be directed to the corresponding author.
